# Hepatic artery aneurysm with no proximal neck and proper hepatic artery bifurcation involvement

**DOI:** 10.1590/1677-5449.202300632

**Published:** 2024-11-15

**Authors:** Pedro Luciano Mellucci, Bruno Aparecido Lourenço de Marqui, Letícia Isper, Adrielle Andrade Pugas, César Alberto Talavera Martelli, Rodolfo Dahlem Melo, Matheus Bertanha, Marcone Lima Sobreira

**Affiliations:** 1 Universidade Estadual Paulista – UNESP, Faculdade de Medicina – FMB, Botucatu, SP, Brasil.; 2 Hospital Regional de Presidente Prudente – HRPP, Presidente Prudente, SP, Brasil.; 3 Universidade do Oeste Paulista – UNOESTE, Presidente Prudente, SP, Brasil.

**Keywords:** hepatic artery, aneurysm, hepatic circulation, vascular surgery

## Abstract

We report the case of a patient with a saccular aneurysm of the hepatic artery with maximum diameter of 2.8 cm, no proximal neck, and involving the bifurcation of the proper hepatic artery, constituting a hostile anatomy for endovascular treatment, which would usually be the first choice for such cases. We performed open surgical treatment with resection and reconstruction using an autologous graft (internal saphenous vein). We illustrate the surgical technique used for adequate vascular exposure of the celiac trunk and hepatic hilum (which is often an area little explored by vascular surgeons) and of structures anatomically close to the hepatic artery. We also illustrate the anastomosis with telescoping technique. We demonstrate the need for vascular surgeons to master the anatomy and classical surgical technique for visceral branches, even in the era of minimally invasive procedures.

## INTRODUCTION

Visceral artery aneurysms (VAAs) are a rare vascular condition, with incidence of 0.01 to 0.2%. They can involve the celiac trunk, renal arteries, and superior or inferior mesenteric arteries and their branches.^1^

Hepatic artery aneurysms (HAAs) account for up to 20% of VAAs and, together with aneurysms of the renal and splenic arteries, are among the most frequent subtypes of this category of aneurysm. While they are frequently asymptomatic, around one in four can rupture, with high documented lethality (up to 40%). They predominantly occur in the sixth decade of life and are more common in males (3:2).^1-4^

Etiology is associated with atherosclerotic disease in up to 62% of cases, but risk factors include hypertension, vasculitis, fibromuscular dysplasia, trauma, surgical procedures, degenerative diseases, infections, vascular connective tissue disease, and congenital anomalies.^5,6^

Considering that the most common presentation of unruptured HAAs is asymptomatic, diagnosis is incidental based on imaging exam findings in the majority of cases. However, this disease can also present with obstructive jaundice, gastrointestinal bleeding, and abdominal pains.^2,5,7^ Diagnosis is generally confirmed with arteriography, computed tomography angiography (angioCT), magnetic resonance angiography, or Doppler ultrasonography.^8^

The complexity of this disease demands correct treatment criteria. Current Society for Vascular Surgery (SVS) treatment guidelines recommend intervention in all hepatic artery pseudoaneurysms, in all symptomatic HAAs, irrespective of size, and in asymptomatic patients with no significant comorbidities and true HAA > 2 cm, or in cases with 0.5 cm/year enlargement. These aneurysms most frequently rupture when diameter is greater than 2 centimeters.^4-6^

Currently available management approaches are open surgery or endovascular repair. Repair is achieved by covered stent placement or coil embolization and is preferably employed for patients with compatible anatomy. Moreover, retrospective studies indicate that the long-term results of open and endovascular surgery are similar, but the morbidity and mortality rates of open repair are significantly higher.^5^

The present study was approved by the Ethics Committee at our institution, under Ethics Appraisal Submission Certificate number 07381519.8.0000.5515 and Research Ethics Committee decision number 5.584.330.

## PART I – CLINICAL SITUATION

The patient was a 73-year-old male Caucasian who was admitted for high-risk unstable angina. On that occasion, he was diagnosed with cardiomyopathy secondary to Chagas disease (ejection fraction = 33%), moderate mitral and tricuspid insufficiency, and three-vessel coronary artery disease and was treated with surgical revascularization by mammary artery bypass to the anterior descending artery.

**Figure 1:**
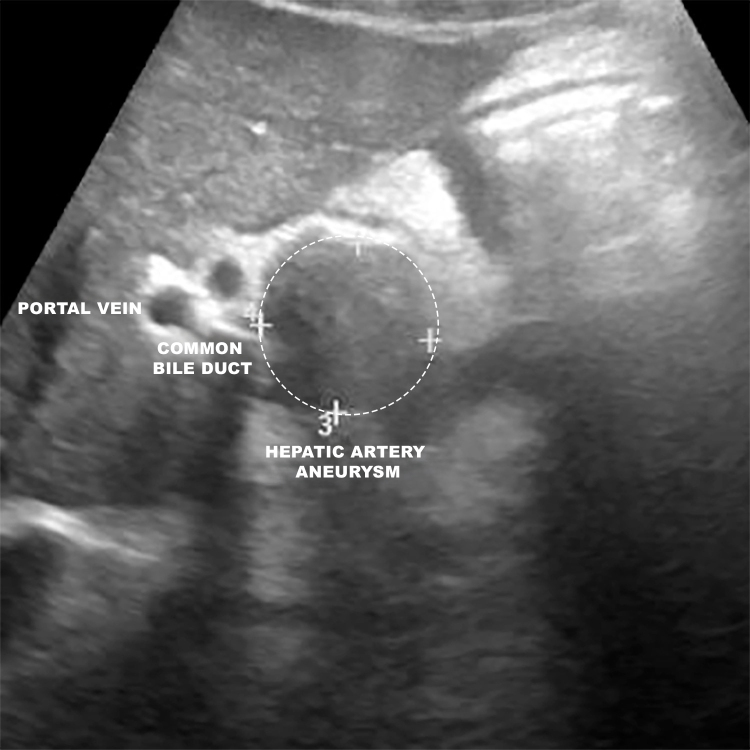
Upper abdominal ultrasound demonstrating a heterogeneous lesion, predominantly echolucent in the topography of the hepatic hilum, with well-defined margins and anatomic correlation with the portal vein, common bile duct, duodenum, and head of the pancreas.

During the admission for cardiovascular treatment, abdominal ultrasonography was performed to investigate nonspecific epigastric pain associated with angina. On this occasion, a heterogeneous nodular formation was observed adjacent to the head of the pancreas (Figure 1). This finding was later confirmed by magnetic resonance with contrast, which revealed a fusiform dilatation of the common hepatic artery and proper hepatic artery, measuring around 3.2 x 2.7 cm at its largest axial diameters, partially thrombosed, and in contact with the anterior wall of the portal vein.

In addition to cardiovascular diseases, the patient’s medical history also included gastroesophageal reflux disease, benign prostate hyperplasia, substernal goiter, arterial hypertension, and dyslipidemia. He had also undergone surgical repair of an intertrochanteric fracture and a cholecystectomy.

## PART II – WHAT WAS DONE

In view of the patient’s high surgical risk, the decision was taken to manage the aneurysm conservatively until the cardiovascular condition had been resolved and then schedule treatment of the HAA.

**Figure 2:**
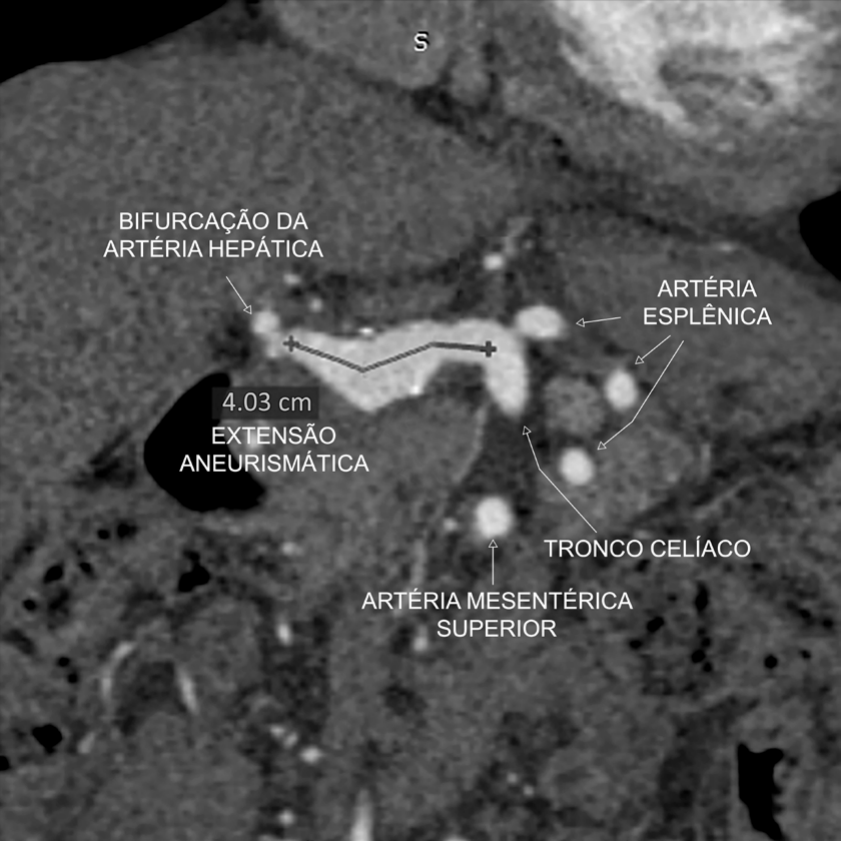
Coronal angiotomography reconstruction showing common and proper hepatic arteries and the caudal saccular dilatation, with total length, with no defined neck at the celiac trunk and involvement of the hepatic bifurcation.

**Figure 3:**
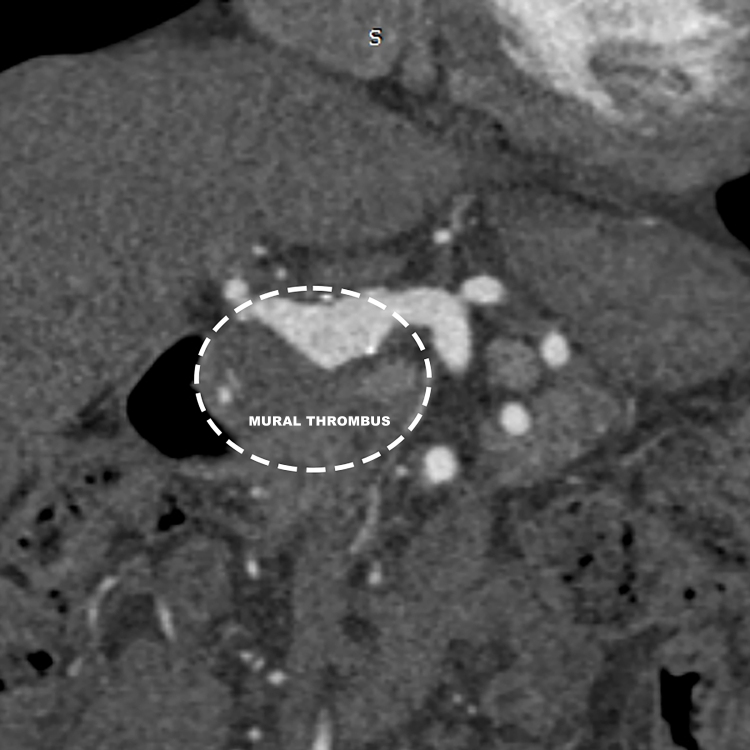
Schematic coronal angiotomography reconstruction, showing the limits of the aneurysm (broken line) and caudal thrombus.

**Figure 4:**
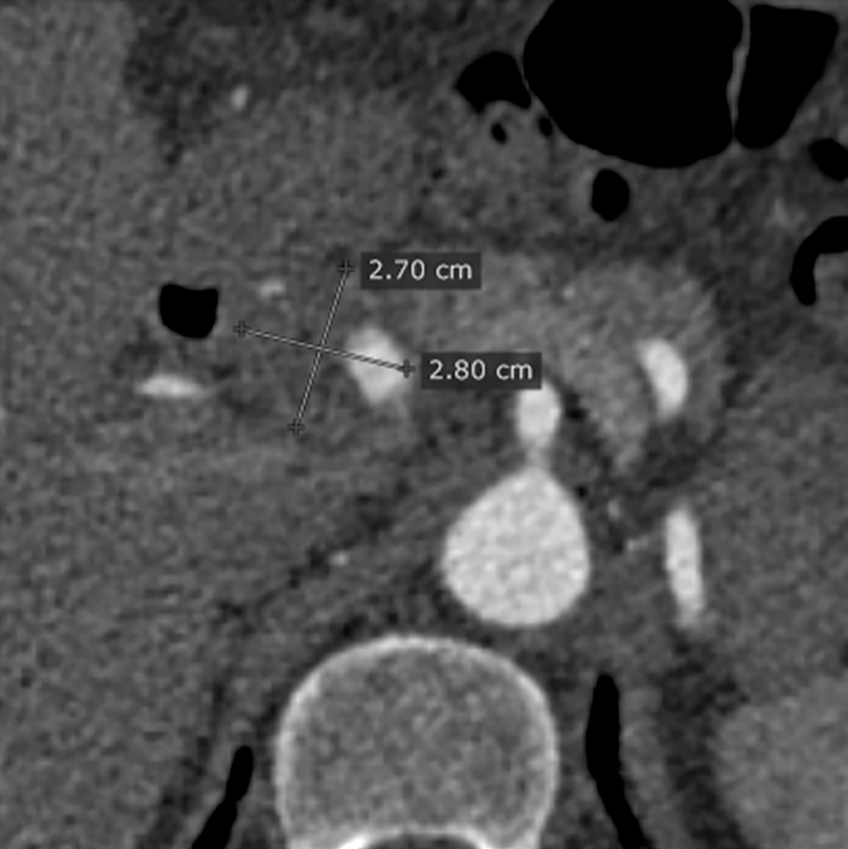
Axial angiotomography image showing the maximum axial diameters of the aneurysm.

**Figure 5:**
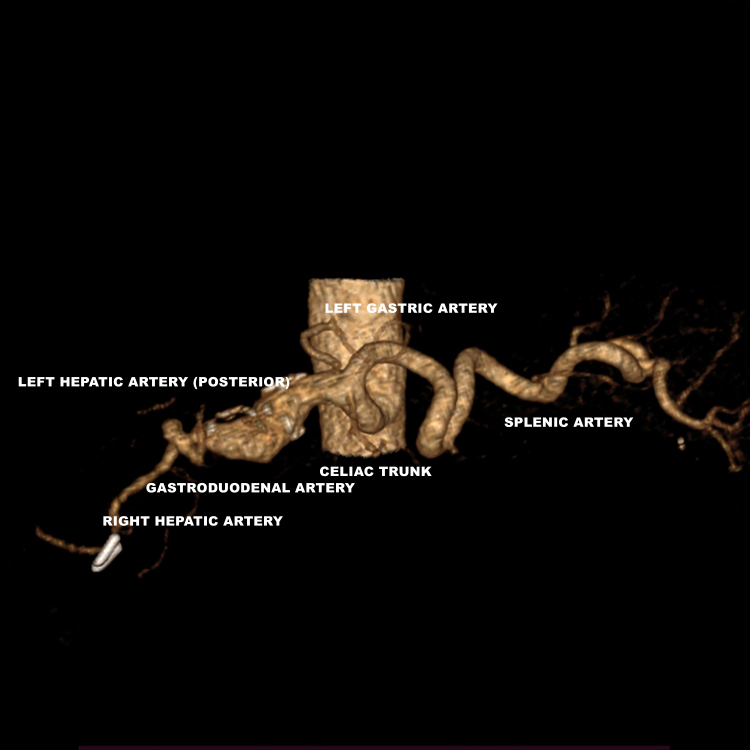
Three-dimensional angiotomography reconstruction in anteroposterior view, showing the anatomy of the region of interest. An artifact can be observed, which is a metal clip from a previous cholecystectomy.

**Figure 6:**
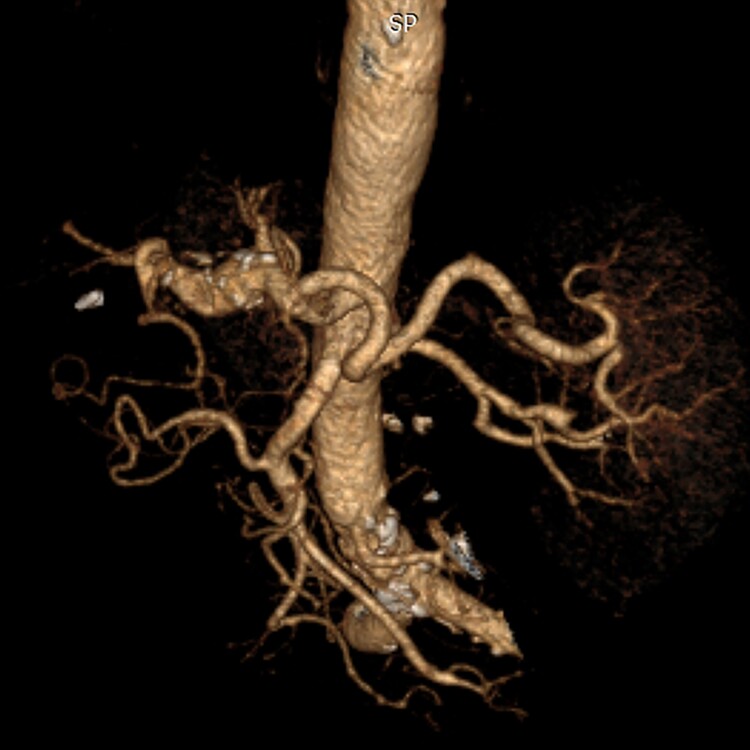
Three-dimensional angiotomography reconstruction in craniocaudal view, showing the anatomy of the region of interest. The lesion appears to have a fusiform format in the three-dimensional reconstruction because of exclusion of the thrombus.

Six months after the cardiovascular treatment, the patient’s aneurysmal disease was staged again with angiotomography. This examination showed an extrahepatic saccular aneurysm of the common and proper hepatic arteries, involving the gastroduodenal artery and the hepatic bifurcation, with no proximal neck at the celiac trunk, a total length of 4.03 cm (Figure 2), mural thrombus (Figure 3), and maximum axial diameters of 2.80 x 2.70 cm (Figure 4). Multiplanar and three-dimensional reconstructions were produced to facilitate treatment planning (Figures 5 and 6).

Initially, the team considered an endovascular approach, because of the patient’s cardiovascular risk. However, the aneurysmal anatomy proved to be overly hostile for coil embolization or covered stenting because of the lack of a proximal neck at the celiac trunk and because of involvement of the hepatic bifurcation. Exclusion of the left hepatic artery could therefore result in significantly higher risk of morbidity and mortality, while deployment of two parallel stents or crushing stent technique present lower patency in smaller caliber arteries. In view of these factors, open HAA repair was planned.

**Figure 7:**
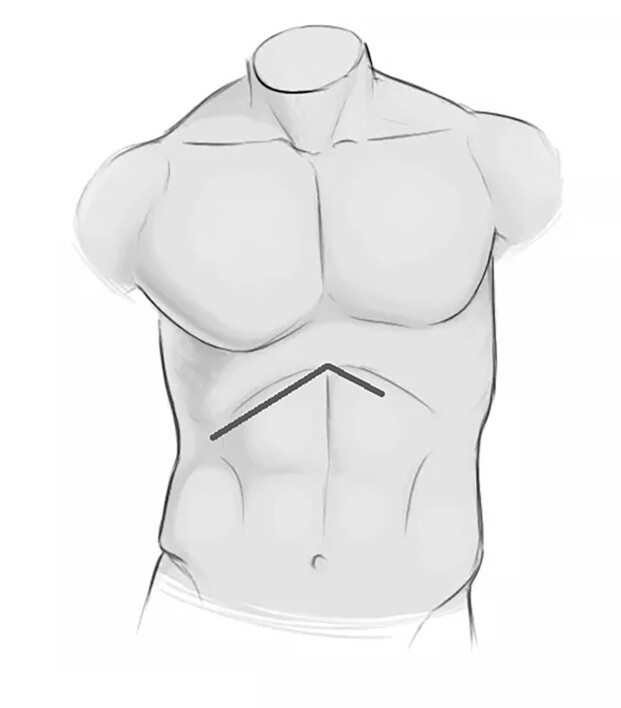
Schematic drawing illustrating the chevron incision used to gain adequate access to the hepatic hilum and celiac trunk.

A Chevron incision was used to obtain adequate surgical exposure of the hepatic hilum and control of the celiac trunk, with a transperitoneal approach via the foramen of Winslow (Figure 7).

**Figure 8:**
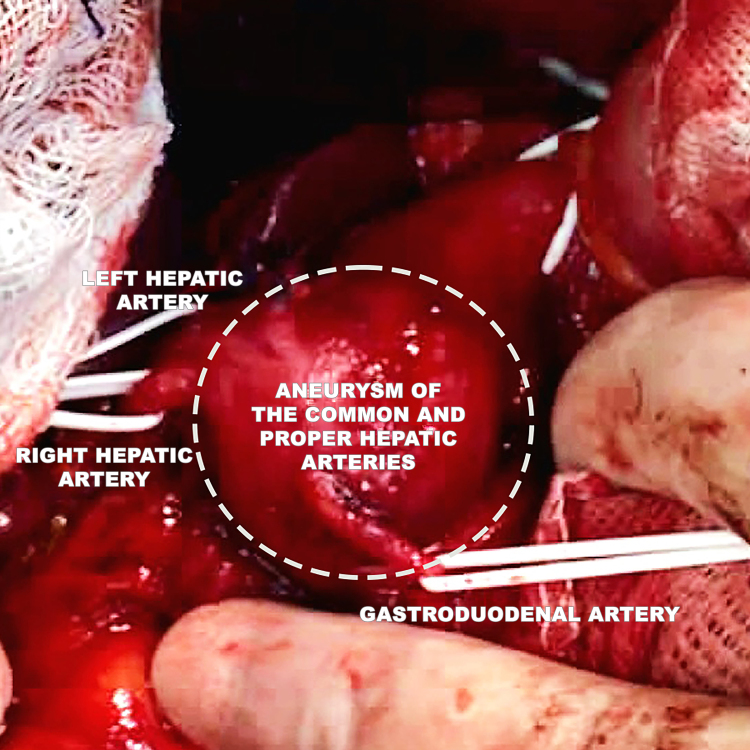
Dissection of the aneurysm (broken line) with isolation of the celiac trunk, right and left hepatic arteries, and gastroduodenal artery.

After opening the foramen of Winslow, proximal control of the celiac trunk was obtained, followed by lateral dissection in the direction of the hepatic hilum and release of the pylorus, duodenum, and the head of the pancreas. After adequate exposure had been achieved, the aneurysm was carefully dissected (Figure 8), with attention to the anatomic relationships posterocaudal to the hepatic artery, especially the portal vein and the common bile duct, which may adhere to the lesion in aneurysms with inflammatory etiology.

**Figure 9:**
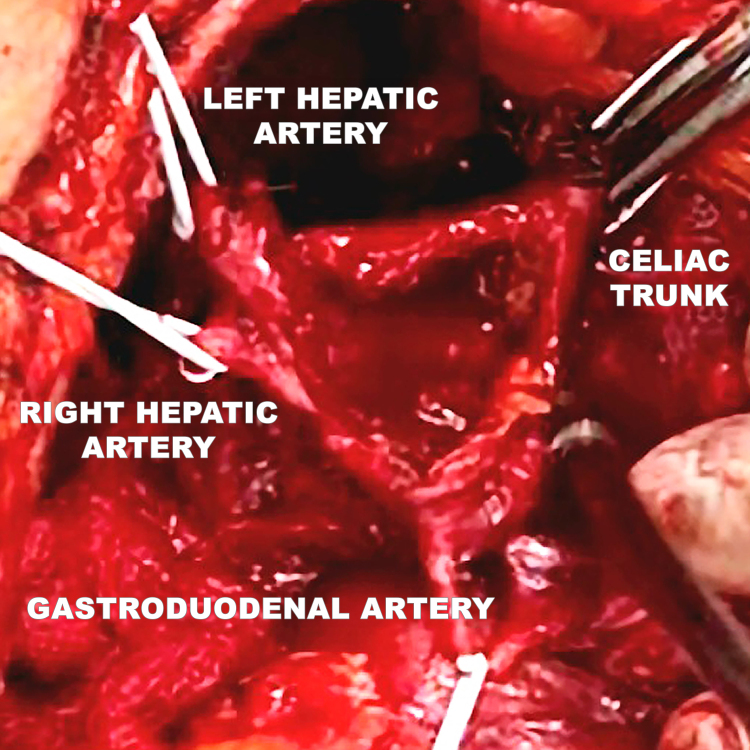
Opening the aneurysm. Proximal clamping at the origin of the hepatic arteries and control of branches with vessel loops and the Potts technique.

After isolation of these arteries, systemic unfractionated heparin was administered in bolus at a dosage of 80 UI/kg. Next, the anterior wall of the aneurysm was opened and the mural thrombus was emptied (Figure 9).

**Figure 10:**
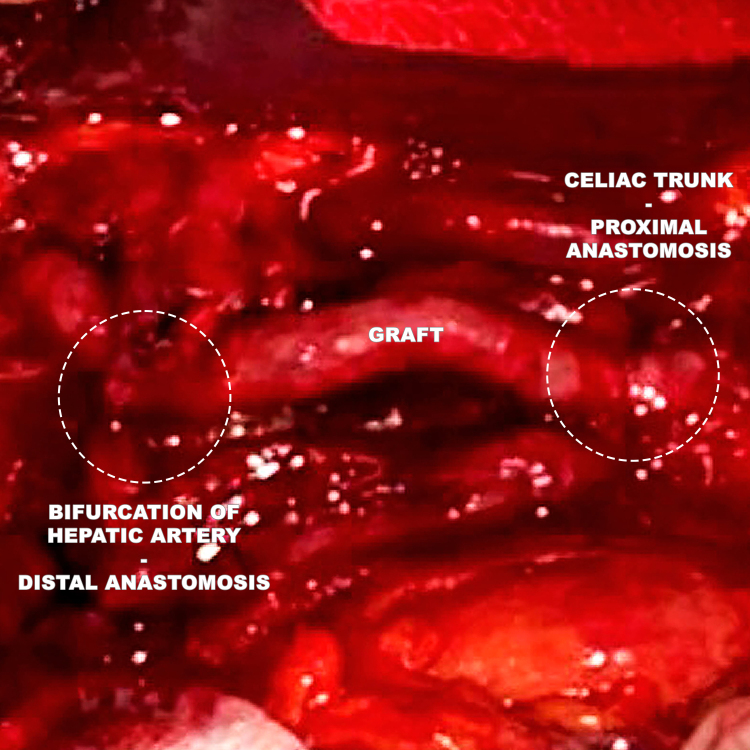
Final appearance, showing the proximal anastomosis with telescoping over the ostium of the hepatic artery, distal anastomosis to both the right and left hepatic arteries, and ligature of the gastroduodenal artery.

The arterial segment was reconstructed with an inverted internal saphenous vein graft by proximal anastomosis, telescoping the venous graft over the celiac trunk at the level of the ostium of the common hepatic artery, where no aneurysmal degeneration was observed. Telescoping is a term imported from digestive surgery, in which one structure involved in an anastomosis is covered by the other. The procedure is performed using separated sutures through both structures in the inside-to-outside direction, reaching a greater depth in the inner structure. This technique is especially useful when there is no adequate proximal neck or exposure is difficult, enabling all of the sutures to be performed separately before the vascular graft is placed at the anastomosis site. The distal anastomosis was constructed over the ostia of the left and right hepatic arteries in conjunction (Figure 10). The gastroduodenal artery had satisfactory reflux and was ligated.

After surgery, the patient was transported to a bed in the intensive care unit (ICU) and was discharged from the ICU after 1 day. The patient was given a test diet on the first postoperative day, which was tolerated well, and he improved steadily thereafter. He was discharged from hospital on the third postoperative day, with no intercurrent conditions, and laboratory test results remained within the limits of normality throughout the hospital stay.

## DISCUSSION

A true aneurysm is a permanent localized dilatation of at least one and a half times the diameter of the vessel, involving all three layers of the vascular wall. Involvement of the hepatic artery is a rare condition, with an estimated incidence of 0.002 to 0.4%.^4,8,9^

Its clinical importance lies in the high mortality rates (25 to 70%) in cases of rupture, reducing perfusion to the hepatobiliary organs and leading to necrosis, hemorrhagic shock, and death. The high mortality rate is linked to the lack of a method for screening for these aneurysms.^9-11^

Possible diagnostic methods for HAA include abdominal ultrasonography, computed tomography, angioCT, magnetic resonance, endoscopy, and angiography. As many reports in the literature show, diagnosis is frequently accidental, as corroborated in the case presented here, in which the patient’s condition was discovered in an ultrasonography examination performed for investigation of epigastric pain. The methods chosen after the ultrasound findings were magnetic resonance with contrast and, afterwards, angioCT for planning surgery with 3D reconstruction, showing anatomic details.^4,11^

The SVS guidelines recommend angioCT as the diagnostic tool of choice for HAAs and mesenteric angiography for preoperative planning. Selective angiography and high resolution angioCT are recommended for assessment of the collateral circulation. It is important to point out that the quality of evidence on which these recommendations are based is level B (moderate).^4^

The options for treatment of HAAs are either open or endovascular surgery. Retrospective studies indicate that the results of repair are similar with both approaches, but morbidity is significantly greater with open repair. ^4^

While endovascular repair is recommended as first-line treatment, certain factors must be considered, such as the aneurysm site, presence of collateral flow, and the patient’s clinical status. Techniques that can be used with this approach include percutaneous embolization or exclusion of the aneurysm with a covered stent. The second of these options is the best technique, but adequate proximal and distal landing zones are needed, meaning it was not feasible for the aneurysm in the case described.^4,6,12^

Although this patient would have been a candidate for the endovascular technique because of his comorbidities, the anatomy of his aneurysm made it impossible to perform this procedure because of the lack of a proximal neck at the celiac trunk and involvement of the hepatic bifurcation.

A recent study indicated that exposure of the hepatic artery via a bilateral subcostal incision is sufficient to expose the infradiaphragmatic aorta, followed by exposure of the trunk of the celiac artery and its branches. That description had several similarities to the approach used in our patient, consisting of a Chevron incision extended transperitoneally in order to reach the celiac trunk and achieve proximal control.^12^

Open surgical options include ligation and exclusion of the aneurysm, providing there is adequate collateral circulation. When collaterals are lacking, aneurysmectomy with reconstructive by-pass or interposition grafting can be employed. Considering that the gastroduodenal artery emerged directly from the aneurysm, after proximal control and isolation of the left and right hepatic arteries and the gastroduodenal artery, an autologous inverted saphenous vein graft was telescoped to the ostium of the hepatic artery over the celiac trunk.^4,12^

Visceral aneurysms, and especially HAAs, tend to be under-diagnosed and are frequently discovered incidentally in routine ultrasonographic examinations. As a result of their low prevalence, current guidelines for treatment decision-making are still based on level low evidence for treatment indications.

Although endovascular treatment is more often chosen for this disease, aneurysms involving arterial bifurcations and absence of a neck constitute a therapeutic challenge, meaning that, even in the era of minimally invasive procedures, the vascular surgeon must master the anatomy and classic surgical techniques of visceral branches.
