# Supplementary material for: Hepatic artery aneurysm with no proximal neck and proper hepatic artery bifurcation involvement
Source: J Vasc Bras. 2024 Nov 15;23:e20230063. doi: 10.1590/1677-5449.202300632 (PMC11614102; doi:10.1590/1677-5449.202300632)
Supplement: Translation

# Translation: Aneurisma de artéria hepática sem colo proximal e acometendo bifurcação de artéria hepática própria

**DOI:** 10.1590/1677-5449.202300631

**Authors:** Pedro Luciano Mellucci, Bruno Aparecido Lourenço de Marqui, Letícia Isper, Adrielle Andrade Pugas, César Alberto Talavera Martelli, Rodolfo Dahlem Melo, Matheus Bertanha, Marcone Lima Sobreira

**Affiliations:** 1 Universidade Estadual Paulista – UNESP, Faculdade de Medicina – FMB, Botucatu, SP, Brasil.; 2 Hospital Regional de Presidente Prudente – HRPP, Presidente Prudente, SP, Brasil.; 3 Universidade do Oeste Paulista – UNOESTE, Presidente Prudente, SP, Brasil.

**Keywords:** artéria hepática, aneurisma, circulação hepática, cirurgia vascular

## Abstract

Apresentamos um relato de caso acerca de um paciente portador de aneurisma sacular de artéria hepática, com diâmetro máximo de 2,8 cm, sem colo proximal e com acometimento da bifurcação da artéria hepática própria, denotando anatomia hostil para tratamento endovascular, que geralmente é a primeira escolha para esses casos. Realizamos tratamento cirúrgico aberto com ressecção e reconstrução a partir de substituto autólogo (veia safena interna). Ilustramos a técnica cirúrgica empregada para a adequada exposição vascular do tronco celíaco e hilo hepático, que muitas vezes é uma área pouco explorada pelo cirurgião vascular, além dos cuidados necessários com estruturas anatomicamente próximas à artéria hepática e da técnica de anastomose com telescopagem. Demonstramos a necessidade de que o cirurgião vascular, mesmo na era dos procedimentos minimamente invasivos, domine a anatomia e a técnica cirúrgica clássica sobre ramos viscerais.

## INTRODUÇÃO

Os aneurismas de artérias viscerais (AAVs) representam uma rara afecção vascular, com incidência entre 0,01 a 0,2%. Os AAVs acometem o tronco celíaco, artérias renais, artérias mesentéricas superior ou inferior e seus ramos^1^ .

Os aneurismas da artéria hepática (AAHs) respondem por até 20% dos AAVs, representando, junto aos aneurismas de artérias renais e esplênica, os mais frequentes dessa classe. Embora frequentemente assintomáticos, cerca de um em cada quatro pode se romper, tendo uma alta letalidade documentada (até 40%). Ocorre predominantemente na sexta década de vida e no sexo masculino (3:2)^1-4^ .

Sua etiologia está associada à doença aterosclerótica em até 62% dos casos, porém hipertensão, vasculites, displasia fibromuscular, trauma, procedimentos cirúrgicos, doenças degenerativas, infecções, doença vascular do colágeno e anomalias congênitas representam fatores de risco^5,6^ .

Considerando que a apresentação mais comum do AAH não roto é assintomática, o seu diagnóstico é incidental através de exame de imagem na maioria das vezes. Entretanto, a doença pode apresentar-se com icterícia obstrutiva, sangramento gastrointestinal e dor abdominal^2,5,7^ . O diagnóstico geralmente é feito através de arteriografia, angiotomografia computadorizada (angioTC), angiorresonância ou ultrassonografia Doppler^8^ .

A complexidade dessa doença exige critérios assertivos de tratamento. As atuais diretrizes da Society for Vascular Surgery (SVS) definem orientações com relação ao seu tratamento, recomendando intervenção em todos os pseudoaneurismas de artéria hepática, em todos os AAHs sintomáticos, independentemente do tamanho, e em pacientes assintomáticos sem comorbidade significativa com AAH verdadeiro > 2 cm ou se houver aumento de 0,5 cm/ano. A ruptura desses aneurismas ocorre mais comumente quando possuem um diâmetro maior que 2 centímetros^4-6^ .

Atualmente, as abordagens disponíveis consistem na cirurgia aberta ou no reparo endovascular. O reparo consiste na terapia com *stent* revestido ou embolização com mola, sendo preferencialmente utilizado em pacientes anatomicamente compatíveis. Ademais, estudos retrospectivos indicam que os resultados a longo prazo para a cirurgia aberta e endovascular são semelhantes, porém a morbimortalidade é significativamente maior no reparo aberto^5^ .

O presente estudo foi aprovado pelo Comitê de Ética de nossa instituição, sob o Certificado de Apresentação de Apreciação Ética número 07381519.8.0000.5515, com o parecer do comitê de ética em pesquisa (CEP) número 5.584.330.

## PARTE I – SITUAÇÃO CLÍNICA

O paciente, caucasiano, do sexo masculino, de 73 anos, foi internado devido a um quadro de angina instável de alto risco. Na ocasião, foi diagnosticado com cardiomiopatia chagásica (fração de ejeção = 33%), insuficiência mitral e tricúspide moderadas e doença arterial coronariana triarterial, sendo submetido a revascularização cirúrgica com ponte mamária para a artéria descendente anterior.

Durante a internação para tratamento cardiovascular, foi realizada ultrassonografia de abdome devido a dor epigástrica inespecífica associada à angina. Nessa ocasião, foi observada uma formação nodular heterogênea adjacente à cabeça do pâncreas (Figura 1), posteriormente confirmada por ressonância contrastada, que revelou uma dilatação fusiforme da artéria hepática comum e própria, medindo cerca de 3,2 x 2,7 cm nos maiores diâmetros axiais, parcialmente trombosada e em contato com a parede anterior da veia porta.

**Figura 1:** Ultrassom de abdome superior demonstrando lesão heterogênea, predominantemente ecolucente em topografia hilar hepática, com limites bem definidos e correlação anatômica com veia porta, colédoco, duodeno e cabeça pancreática.

Como antecedentes pessoais, além das doenças cardiovasculares, apresentava doença do refluxo gastroesofágico, hiperplasia prostática benigna, bócio mergulhante, hipertensão arterial e dislipidemia. Ademais, apresentava antecedente cirúrgico de correção de fratura transtrocantérica e colecistectomia.

## PARTE II – O QUE FOI FEITO

Considerando o alto risco cirúrgico do paciente, optou-se por uma conduta conservadora até a resolução do quadro cardiovascular, com posterior programação para o tratamento do AAH.

Após 6 meses do tratamento cardiovascular, foi realizado reestadiamento da doença aneurismática através de angiotomografia. O exame demonstrou um aneurisma extra-hepático sacular das artérias hepáticas comum e própria, acometendo a artéria gastroduodenal e a bifurcação hepática, sem colo proximal com o tronco celíaco, com extensão total de 4,03 cm (Figura 2), trombo mural (Figura 3) e diâmetros axiais máximos de 2,80 x 2,70 cm (Figura 4). Reconstruções multiplanares e tridimensionais foram realizadas a fim de auxiliar no planejamento terapêutico (Figuras 5 e 6).

**Figura 2:** Angiotomografia em reconstrução coronal demonstrando a artéria hepática comum e própria, assim como a dilação sacular caudal e sua extensão total, sem colo definido com o tronco celíaco e acometimento da bifurcação hepática.

**Figura 3:** Angiotomografia em reconstrução coronal esquemática, com delimitação de limites da lesão aneurismática (tracejado) e trombo caudal.

**Figura 4:** Angiotomografia em corte axial demonstrando diâmetros axiais máximos da lesão aneurismática.

**Figura 5:** Angiotomografia com reconstrução tridimensional em visão anteroposterior demonstrando anatomia da região de interesse. Observa-se artefato de *clip* metálico devido a colecistectomia prévia.

**Figura 6:** Angiotomografia com reconstrução tridimensional em visão craniocaudal demonstrando anatomia da região de interesse. A lesão apresenta conformação fusiforme na reconstrução tridimensional devido à exclusão do trombo.

Inicialmente, a equipe considerou a abordagem endovascular devido ao alto risco cardiovascular do paciente. A anatomia da lesão aneurismática, entretanto, demonstrou-se hostil para a embolização com *coils* ou uso de *stents* recobertos devido à ausência de colo proximal com o tronco celíaco, assim como ao acometimento da bifurcação hepática. Dessa forma, a exclusão da hepática esquerda poderia acarretar um aumento expressivo da morbimortalidade, enquanto o uso de dois *stents* paralelos ou técnica de *crushing stent* apresenta baixa perviedade em artérias de calibre reduzido. Assim, optou-se pela abordagem aberta do AAH.

Para uma exposição cirúrgica adequada do hilo hepático e controle do tronco celíaco, optou-se por uma incisão de Chevron, com abordagem transperitoneal a partir do forame de Winslow (Figura 7).

**Figura 7:** Desenho esquemático demonstrando incisão de Chevron para o adequado acesso hepático hilar e do tronco celíaco.

A partir da abertura do forame de Winslow, realizou-se o controle proximal do tronco celíaco, dissecção lateral em direção ao hilo hepático e rebatimento de piloro, duodeno e cabeça pancreática. Após a exposição adequada, foi realizada uma dissecção cuidadosa (Figura 8) da lesão aneurismática, com atenção às relações anatômicas posterocaudais da artéria hepática, especialmente a veia porta e o ducto colédoco, que podem apresentar-se aderidos à lesão em aneurismas de etiologia inflamatória.

**Figura 8:** Dissecção da lesão aneurismática (tracejado) com isolamento de tronco celíaco, artérias hepáticas direita e esquerda e artéria gastroduodenal.

Após o isolamento das artérias em questão, foi administrado um *bolus* de heparina não fracionada sistêmica na dose de 80 UI/kg. Em seguida, procedeu-se com a abertura da parede anterior da lesão e o esvaziamento do trombo mural (Figura 9).

**Figura 9:** Abertura da lesão aneurismática. Clampeamento proximal na emergência hepática e controle de ramos com *vessel loops* em alças de Potts.

Para a reconstrução do segmento arterial, foi realizada enxertia com veia safena interna invertida a partir de anastomose proximal por telescopagem do enxerto venoso sobre o tronco celíaco ao nível do óstio da artéria hepática comum, onde não se observava degeneração aneurismática. A telescopagem é um termo importado da cirurgia do aparelho digestivo, onde, em uma anastomose, uma estrutura é englobada pela outra. Esse procedimento é realizado com pontos separados no sentido dentro-fora em ambas as estruturas, atingindo uma maior profundidade na estrutura que será interiorizada. Essa técnica é especialmente útil na ausência de um colo proximal adequado ou dificuldade de exposição, permitindo que todos os pontos sejam realizados separadamente antes que o substituto vascular seja levado até o leito de anastomose. A anastomose distal foi realizada sobre o óstio das artérias hepáticas direita e esquerda de forma conjunta (Figura 10). A artéria gastroduodenal apresentava refluxo satisfatório e foi ligada.

**Figura 10:** Aspecto final, onde observa-se anastomose proximal a partir de telescopagem sobre o óstio da artéria hepática, anastomose distal comum em hepáticas direita e esquerda e ligadura de artéria gastroduodenal.

Após o tratamento cirúrgico, o paciente foi encaminhado para um leito de unidade de terapia intensiva (UTI), recebendo alta da UTI após um dia. Recebeu dieta de prova no primeiro dia pós-operatório, com boa tolerância e evolução subsequente. Por fim, recebeu alta hospitalar no terceiro dia pós-operatório, sem intercorrências, mantendo exames laboratoriais dentro dos limites da normalidade durante a internação.

## DISCUSSÃO

O aneurisma verdadeiro consiste na dilatação permanente e localizada de, no mínimo, uma vez e meia o diâmetro do vaso, envolvendo as três camadas da parede vascular. O acometimento da artéria hepática consiste em uma afecção rara, com incidência estimada de 0,002 a 0,4%^4,8,9^ .

Sua importância clínica está relacionada às altas taxas de mortalidade (25 a 70%) em casos de ruptura, resultando na diminuição da perfusão dos órgãos hepatobiliares, necrose, choque hemorrágico e morte. Essa alta taxa de fatalidade está atrelada à inexistência de um método de triagem para esse aneurisma^9-11^ .

Os possíveis métodos diagnósticos do AAH incluem ultrassonografia abdominal, tomografia computadorizada, angioTC, ressonância magnética, endoscopia e angiografia. Como muitas literaturas relatam, o diagnóstico é frequentemente acidental, como comprovado no paciente apresentado, cuja condição foi descoberta através de uma ultrassonografia para investigação de dor epigástrica. Os métodos escolhidos subsequencialmente foram a ressonância magnética contrastada e, posteriormente, a angioTC para programação cirúrgica e reconstrução 3D, visando detalhamento anatômico^4,11^ .

As diretrizes da SVS recomendam a angioTC como ferramenta diagnóstica de escolha para os AAHs e a angiografia mesentérica para o planejamento pré-operatório. Para a avaliação da circulação colateral, recomenda-se a angiografia seletiva e a angioTC de alta resolução. É importante ressaltar que a qualidade de evidência foi nível B (moderado) para tais recomendações^4^ .

As possibilidades para o tratamento dos AAHs são cirurgia aberta ou endovascular. Estudos retrospectivos indicaram que os resultados após o reparo de ambas abordagens são semelhantes, no entanto, a morbidade é significativamente maior no reparo aberto^4^ .

Embora o reparo endovascular seja recomendado como tratamento de primeira linha, alguns fatores devem ser considerados, como a localização do aneurisma, a presença de fluxo colateral e o estado clínico do paciente. Ademais, as possíveis técnicas dessa abordagem incluem a embolização percutânea ou a exclusão do aneurisma através de um *stent* coberto. Essa última abordagem consiste na melhor técnica descrita, porém requer zonas de vedação proximal e distal adequadas, o que não era viável no aneurisma apresentado no caso^4,6,12^ .

Apesar do paciente ser candidato à técnica endovascular devido às suas comorbidades, a anatomia de seu aneurisma impossibilitou que esse procedimento fosse realizado, atentando-se à ausência de colo proximal com o tronco celíaco e ao acometimento da bifurcação hepática.

Estudos recentes indicaram que a exposição da artéria hepática através de incisão subcostal bilateral é adequada na exposição da aorta infradiafragmática, seguida pela exposição do tronco da artéria celíaca e seus ramos. A descrição apresentou diversas semelhanças com a abordagem adotada em nosso paciente, consistindo na incisão de Chevron, com avanço transperitoneal no intuito de alcançar o tronco celíaco para o controle proximal^12^_._

As opções cirúrgicas abertas incluem a ligadura e exclusão do aneurisma, desde que haja colaterais adequadas. Na ausência dessas, a aneurismectomia com reconstrução via *by-pass* ou enxerto de interposição podem ser utilizados. Considerando a emergência da artéria gastroduodenal diretamente do aneurisma, após o controle proximal e isolamento das artérias hepática direita e esquerda e artéria gastroduodenal, optou-se por um enxerto de veia safena autóloga invertida, telescopando-a ao óstio da artéria hepática sobre o tronco celíaco^4,12^_._

Os aneurismas viscerais, especialmente o AAH, costumam ser subdiagnosticados e frequentemente descobertos de forma incidental em exames ultrassonográficos de rotina. Devido à sua baixa prevalência, as diretrizes atuais para a decisão terapêutica ainda apresentam um baixo nível de evidência quanto à indicação do tratamento.

Embora a terapia endovascular seja mais comumente indicada para essa doença, lesões que acometem bifurcações arteriais e ausência de colo podem apresentar-se como um desafio terapêutico, trazendo à tona a necessidade do cirurgião vascular, mesmo na era dos procedimentos minimamente invasivos, dominar a anatomia e a técnica cirúrgica clássica sobre ramos viscerais.
